# Supplementary material for: Talking About Weight with Children: Associations with Parental Stigma, Bias, Attitudes, and Child Weight Status
Source: Nutrients. 2025 Sep 10;17(18):2920. doi: 10.3390/nu17182920 (PMC12472602; doi:10.3390/nu17182920)
Supplement: Supplementary file 1 [file nutrients-17-02920-s001.zip › nutrients-3815454-supplementary.pdf]

Supplementary Table S1: Description and Coding of Study Variables.

Variable / Construct	Description & Items	Coding Scheme and Scale
**Communication Variables**		
**Health-Focused Conversations**	Mean of 2 items asking about the frequency of conversations about (a) healthy eating habits and (b) being physically active.	Scale: 1 (Never or rarely) to 5 (Almost every day).
**Weight-Focused Conversations**	Mean of 4 items asking about the frequency of conversations about the child's weight/size, telling the child they weigh too much, suggesting diet changes for weight reasons, and recommending exercise for weight management.	Scale: 1 (Never or rarely) to 5 (Almost every day).
**Comments on Parent's Own Weight**	Single item assessing the frequency of parental comments made about their own weight, shape, or size.	Scale: 1 (Never), 2 (Rarely), 3 (Sometimes), 4 (Often/Very often).
**Comments on Others' Weight**	Single item assessing the frequency of parental comments made about other people's weight, shape, or size.	Scale: 1 (Never), 2 (Rarely), 3 (Sometimes), 4 (Often/Very often).
**Mentions of Diet/Exercise Routines**	Single item assessing the frequency of parental mentions of their own dieting or exercise routines.	Scale: 1 (Never), 2 (Rarely), 3 (Sometimes), 4 (Often/Very often).
**Stigma, Bias, and Attitude Variables**		
**Experienced Weight Stigma**	A history of having experienced weight-based teasing or unfair treatment.	Computation: Composite binary variable coded 1 if participant answered 'yes' to being teased OR treated unfairly due to weight.<br>Scale: 0 (No), 1 (Yes).
**Internalized Weight Bias**	Mean score on the 10-item modified Weight Bias Internalization Scale (WBIS-M), assessing self-devaluation due to weight.	Scale: 1 (Strongly disagree) to 7 (Strongly agree).
**Antifat Attitudes**	Mean score on the 13-item Antifat Attitudes Questionnaire (AFA), assessing explicit negative attitudes toward fatness.	Scale: 0 (Very strongly disagree) to 9 (Extremely agree).
**Universal Measure of Bias**	Total score on the 20-item Universal Measure of Bias (Fat Phobia Scale), assessing attitudes towards people with obesity.	Computation: Sum of 20 items. Specific items were reverse-coded for consistency.<br>Scale: Possible score range 20-140.
**Dichotomized Covariates for Regression**		
**Parent Age Group**	Parent's age categorized for use in regression models.	0 = <40 years<br>1 = ≥ 40 years
**Parent Education**	Parent's highest educational attainment, categorized.	0 = Lower Education (Primary through Post-secondary)<br>1 = Higher Education (Bachelor's degree or higher)
**Marital Status**	Parent's marital status, categorized.	0 = Divorced/Widowed/Never Married<br>1 = Married

**Self-Rated Health**	Parent's self-perception of their general health, categorized.	0 = Very Poor/Poor/Acceptable<br>1 = Good/Very Good
**Number of Children**	Family size, categorized.	0 = One child<br>1 = More than one child
**Parent BMI Status**	Parent's Body Mass Index classification, categorized.	0 = Underweight/Normal weight<br>1 = Overweight/Obese
**Child Weight Status**	Child's weight status based on WHO percentiles, categorized.	0 = Underweight/Healthy Weight<br>1 = Overweight/Obese

Supplementary Table S2: Item-Level Distribution of Responses to the child-centered weight and health conversations and weight comments about oneself and others

Item	Category	n	%	Valid %
Frequency of healthy eating conversations with child	Never or rarely	1	0.2	0.2
	Once in a while	30	7.2	7.2
	Sometimes	76	18.4	18.4
	Often	225	54.3	54.3
	Almost every day	82	19.8	19.8
Frequency of physical activity conversations with child	Never or rarely	2	0.5	0.5
	Once in a while	33	8	8
	Sometimes	64	15.5	15.5
	Often	253	61.1	61.1
	Almost every day	62	15	15
Frequency of conversations regarding child's weight or body size	Never or rarely	19	4.6	4.6
	Once in a while	65	15.7	15.7
	Sometimes	147	35.5	35.5
	Often	157	37.9	37.9
	Almost every day	26	6.3	6.3
Frequency of indicating that child weighs too much	Never or rarely	255	61.6	61.6
	Once in a while	58	14	14
	Sometimes	58	14	14
	Often	35	8.5	8.5
	Almost every day	8	1.9	1.9
Frequency of suggesting dietary changes for weight loss	Never or rarely	236	57	57
	Once in a while	54	13	13
	Sometimes	56	13.5	13.5
	Often	54	13	13
	Almost every day	14	3.4	3.4
Frequency of recommending exercise for weight management	Never or rarely	164	39.6	39.6
	Once in a while	69	16.7	16.7
	Sometimes	81	19.6	19.6
	Often	83	20	20
	Almost every day	17	4.1	4.1
Frequency of parental comments about own weight, shape, or size based on child's perceived weight status	Never	42	10.1	10.1
	Rarely	105	25.4	25.4
	Sometimes	184	44.4	44.4
	Often/Very often	83	20	20
Frequency of parental comments about others' weight, shape, or size in the presence of child	Never	137	33.1	33.1
	Rarely	159	38.4	38.4
	Sometimes	96	23.2	23.2
	Often/Very often	22	5.3	5.3
Frequency of parental mentions of own dieting or exercise routines with child	Never	35	8.5	8.5
	Rarely	113	27.3	27.3
	Sometimes	181	43.7	43.7
	Often/Very often	85	20.5	20.5

Supplementary Table S3: Item-Level Distribution of Responses to the modified weight bias internalization scale (WBIS-M);

**Item (Variable Name)**	**Response Category**	**n**	**%**
(IWB1): Level of agreement that the respondent does not deserve a fulfilling social life because of their weight	Strongly disagree	275	66.4
	Disagree	78	18.8
	Somewhat disagree	13	3.1
	Neither agree nor disagree	24	5.8
	Agree	15	3.6
	Strongly agree	9	2.2
(IWB2): Level of agreement that the respondent is less attractive than most others because of their weight	Strongly disagree	198	47.8
	Disagree	98	23.7
	Somewhat disagree	38	9.2
	Neither agree nor disagree	37	8.9
	Agree	37	8.9
	Strongly agree	6	1.4
(IWB3): Level of agreement that the respondent feels anxious about being with others because of their weight	Strongly disagree	212	51.2
	Disagree	92	22.2
	Somewhat disagree	36	8.7
	Neither agree nor disagree	24	5.8
	Agree	41	9.9
	Strongly agree	9	2.2
(IWB4): Level of agreement that the respondent does not feel able to fully enjoy life because of their weight	Strongly disagree	225	54.3
	Disagree	93	22.5
	Somewhat disagree	27	6.5
	Neither agree nor disagree	24	5.8
	Agree	29	7
	Strongly agree	16	3.9
(IWB5): Level of agreement that the respondent feels less worthy than others because of their weight	Strongly disagree	287	69.3
	Disagree	81	19.6
	Somewhat disagree	11	2.7
	Neither agree nor disagree	22	5.3
	Agree	9	2.2
	Strongly agree	4	1
(IWB6): Level of agreement that the respondent feels frustrated with themselves because of their weight	Strongly disagree	197	47.6
	Disagree	87	21
	Somewhat disagree	47	11.4
	Neither agree nor disagree	21	5.1
	Agree	46	11.1
	Strongly agree	16	3.9
(IWB7): Level of agreement that the respondent does not deserve good things in life because of their weight	Strongly disagree	297	71.7
	Disagree	73	17.6
	Somewhat disagree	20	4.8
	Neither agree nor disagree	13	3.1
	Agree	5	1.2
	Strongly agree	6	1.4
(IWB8): Level of agreement that the respondent feels ashamed of themselves because of their weight	Strongly disagree	251	60.6
	Disagree	84	20.3
	Somewhat disagree	25	6
	Neither agree nor disagree	25	6
	Agree	24	5.8
	Strongly agree	5	1.2
	Strongly disagree	283	68.4

(IWB9): Level of agreement that the respondent feels disrespected by others because of their weight	Disagree	80	19.3
	Somewhat disagree	21	5.1
	Neither agree nor disagree	18	4.3
	Agree	11	2.7
	Strongly agree	1	0.2
(IWB10): Level of agreement that the respondent feels upset when thinking about their weight	Strongly disagree	206	49.8
	Disagree	84	20.3
	Somewhat disagree	36	8.7
	Neither agree nor disagree	25	6
	Agree	46	11.1
	Strongly agree	17	4.1

Note. N=414. WBIS-M uses a 7-point Likert scale (1=Strongly disagree to 7=Strongly agree). Higher mean scores indicate greater internalized weight bias.

Supplementary Table S4: Item-Level Distribution of Responses to the The Antifat Attitudes Questionnaire (AFA);

Item (Variable Name & Subscale)	Response Category	n	%
(AFA1 - Dislike): I really don't like fat people much	Very strongly disagree	190	45.9
	Strongly disagree	42	10.1
	Disagree	63	15.2
	Slightly disagree	17	4.1
	Neither agree nor disagree	52	12.6
	Slightly agree	27	6.5
	Agree	15	3.6
	Strongly agree	3	0.7
	Very strongly agree	2	0.5
	Extremely agree	3	0.7
(AFA2 - Dislike): I don't have many friends that are fat	Very strongly disagree	91	22
	Strongly disagree	26	6.3
	Disagree	60	14.5
	Slightly disagree	34	8.2
	Neither agree nor disagree	69	16.7
	Slightly agree	35	8.5
	Agree	58	14
	Strongly agree	15	3.6
	Very strongly agree	11	2.7
	Extremely agree	15	3.6
(AFA3 - Dislike): I tend to think that overweight people are a little untrustworthy	Very strongly disagree	77	18.6
	Strongly disagree	27	6.5
	Disagree	56	13.5
	Slightly disagree	28	6.8
	Neither agree nor disagree	64	15.5
	Slightly agree	72	17.4
	Agree	62	15
	Strongly agree	16	3.9
	Very strongly agree	3	0.7
	Extremely agree	9	2.2
(AFA4 - Dislike): I think fat people tend not to be as bright as normal weight people	Very strongly disagree	226	54.6
	Strongly disagree	43	10.4
	Disagree	52	12.6
	Slightly disagree	19	4.6
	Neither agree nor disagree	42	10.1
	Slightly agree	12	2.9
	Agree	14	3.4
	Strongly agree	1	0.2
	Very strongly agree	3	0.7
	Extremely agree	2	0.5
(AFA5 - Dislike): I have a hard time taking fat people too seriously	Very strongly disagree	252	60.9
	Strongly disagree	50	12.1
	Disagree	61	14.7
	Slightly disagree	10	2.4
	Neither agree nor disagree	26	6.3
	Slightly agree	8	1.9
	Agree	3	0.7

	Strongly agree	2	0.5
	Very strongly agree	2	0.5
	Extremely agree	0	0
(AFA6 - Dislike): I feel somewhat uncomfortable around fat people	Very strongly disagree	237	57.2
	Strongly disagree	51	12.3
	Disagree	57	13.8
	Slightly disagree	23	5.6
	Neither agree nor disagree	25	6
	Slightly agree	13	3.1
	Agree	3	0.7
	Strongly agree	2	0.5
	Very strongly agree	0	0
	Extremely agree	3	0.7
(AFA7 - Dislike): I might avoid hiring a fat person if I were an employer	Very strongly disagree	234	56.5
	Strongly disagree	56	13.5
	Disagree	59	14.3
	Slightly disagree	16	3.9
	Neither agree nor disagree	25	6
	Slightly agree	11	2.7
	Agree	4	1
	Strongly agree	4	1
	Very strongly agree	3	0.7
	Extremely agree	2	0.5
(AFA8 - Fear of Fat): I feel disgusted with myself when I gain weight	Very strongly disagree	171	41.3
	Strongly disagree	45	10.9
	Disagree	66	15.9
	Slightly disagree	34	8.2
	Neither agree nor disagree	40	9.7
	Slightly agree	25	6
	Agree	15	3.6
	Strongly agree	6	1.4
	Very strongly agree	3	0.7
	Extremely agree	9	2.2
(AFA9 - Fear of Fat): One of the worst things that could happen to me would be gaining 25 pounds	Very strongly disagree	103	24.9
	Strongly disagree	35	8.5
	Disagree	47	11.4
	Slightly disagree	30	7.2
	Neither agree nor disagree	45	10.9
	Slightly agree	39	9.4
	Agree	43	10.4
	Strongly agree	23	5.6
	Very strongly agree	20	4.8
	Extremely agree	29	7
(AFA10 - Fear of Fat): I worry about becoming fat	Very strongly disagree	76	18.4
	Strongly disagree	45	10.9
	Disagree	52	12.6
	Slightly disagree	33	8
	Neither agree nor disagree	38	9.2
	Slightly agree	54	13

	Agree	55	13.3
	Strongly agree	25	6
	Very strongly agree	14	3.4
	Extremely agree	22	5.3
(AFA11 - Willpower): Overweight people can lose weight with a little exercise	Very strongly disagree	45	10.9
	Strongly disagree	30	7.2
	Disagree	27	6.5
	Slightly disagree	28	6.8
	Neither agree nor disagree	47	11.4
	Slightly agree	46	11.1
	Agree	92	22.2
	Strongly agree	27	6.5
	Very strongly agree	36	8.7
	Extremely agree	36	8.7
(AFA12 - Willpower): Some people are fat because they have no willpower	Very strongly disagree	74	17.9
	Strongly disagree	34	8.2
	Disagree	38	9.2
	Slightly disagree	32	7.7
	Neither agree nor disagree	55	13.3
	Slightly agree	63	15.2
	Agree	43	10.4
	Strongly agree	27	6.5
	Very strongly agree	21	5.1
	Extremely agree	27	6.5
(AFA13 - Willpower): Fat people tend to be fat largely through their own fault	Very strongly disagree	81	19.6
	Strongly disagree	34	8.2
	Disagree	54	13
	Slightly disagree	36	8.7
	Neither agree nor disagree	66	15.9
	Slightly agree	58	14
	Agree	34	8.2
	Strongly agree	16	3.9
	Very strongly agree	18	4.3
	Extremely agree	17	4.1

Note. N=414. AFA uses a 10-point scale (0=Very strongly disagree to 9=Extremely agree). Higher mean scores indicate stronger antifat attitudes.

Supplementary Table S5: Item-Level Distribution of Responses to the Universal Measure of Bias Scale (UMBFAT)

Item (Variable Name & Description from Output)	Response Category	n	%
(UMB1) Agreement that people with obesity tend toward bad behaviour (reverse-coded)	Strongly agree	184	44.4
	Moderately agree	33	8
	Slightly agree	29	7
	Neither agree nor disagree	79	19.1
	Slightly disagree	37	8.9
	Moderately disagree	12	2.9
	Strongly disagree	40	9.7
(UMB2) Agreement that people with obesity are sloppy (reverse-coded)	Strongly agree	107	25.8
	Moderately agree	53	12.8
	Slightly agree	37	8.9
	Neither agree nor disagree	83	20
	Slightly disagree	64	15.5
	Moderately disagree	29	7
	Strongly disagree	41	9.9
(UMB3) Agreement that people with obesity are dishonest (reverse-coded)	Strongly agree	222	53.6
	Moderately agree	32	7.7
	Slightly agree	21	5.1
	Neither agree nor disagree	70	16.9
	Slightly disagree	21	5.1
	Moderately disagree	4	1
	Strongly disagree	44	10.6
(UMB4) Agreement that people with obesity have bad hygiene (reverse-coded)	Strongly agree	198	47.8
	Moderately agree	40	9.7
	Slightly agree	28	6.8
	Neither agree nor disagree	70	16.9
	Slightly disagree	22	5.3
	Moderately disagree	14	3.4
	Strongly disagree	42	10.1
(UMB5) Agreement that people with obesity do not consider the needs of others (reverse-coded)	Strongly agree	215	51.9
	Moderately agree	39	9.4
	Slightly agree	18	4.3
	Neither agree nor disagree	71	17.1
	Slightly disagree	23	5.6
	Moderately disagree	6	1.4
	Strongly disagree	42	10.1
6 (UMB6) Agreement that one would not want a person with obesity as a roommate (reverse-coded)	Strongly agree	208	50.2
	Moderately agree	37	8.9
	Slightly agree	26	6.3
	Neither agree nor disagree	61	14.7
	Slightly disagree	21	5.1
	Moderately disagree	16	3.9
	Strongly disagree	45	10.9
(UMB7) Agreement that one likes people with obesity	Strongly agree	47	11.4
	Moderately agree	25	6
	Slightly agree	24	5.8
	Neither agree nor disagree	193	46.6
	Slightly disagree	33	8

	Moderately disagree	29	7
	Strongly disagree	63	15.2
(UMB8) Agreement that one does not enjoy having a conversation with a person with obesity (reverse-coded)	Strongly agree	223	53.9
	Moderately agree	34	8.2
	Slightly agree	22	5.3
	Neither agree nor disagree	62	15
	Slightly disagree	18	4.3
	Moderately disagree	6	1.4
	Strongly disagree	49	11.8
(UMB9) Agreement that one would be comfortable having a person with obesity among friends	Strongly agree	130	31.4
	Moderately agree	28	6.8
	Slightly agree	30	7.2
	Neither agree nor disagree	126	30.4
	Slightly disagree	23	5.6
	Moderately disagree	17	4.1
	Strongly disagree	60	14.5
(UMB10) Agreement that one would like having a person with obesity at one's place of worship or community centre	Strongly agree	103	24.9
	Moderately agree	26	6.3
	Slightly agree	33	8
	Neither agree nor disagree	175	42.3
	Slightly disagree	25	6
	Moderately disagree	13	3.1
	Strongly disagree	39	9.4
(UMB11) Agreement that one finds people with obesity attractive	Strongly agree	45	10.9
	Moderately agree	29	7
	Slightly agree	35	8.5
	Neither agree nor disagree	172	41.5
	Slightly disagree	33	8
	Moderately disagree	22	5.3
	Strongly disagree	78	18.8
(UMB12) Agreement that people with obesity make good romantic partners	Strongly agree	36	8.7
	Moderately agree	26	6.3
	Slightly agree	26	6.3
	Neither agree nor disagree	236	57
	Slightly disagree	29	7
	Moderately disagree	18	4.3
	Strongly disagree	43	10.4
(UMB13) Agreement that one finds people with obesity sexy	Strongly agree	31	7.5
	Moderately agree	21	5.1
	Slightly agree	28	6.8
	Neither agree nor disagree	183	44.2
	Slightly disagree	26	6.3
	Moderately disagree	31	7.5
	Strongly disagree	94	22.7
(UMB14) Agreement that people with obesity are a turn-off (reverse-coded)	Strongly agree	187	45.2
	Moderately agree	30	7.2
	Slightly agree	20	4.8
	Neither agree nor disagree	96	23.2
	Slightly disagree	21	5.1

	Moderately disagree	14	3.4
	Strongly disagree	46	11.1
(UMB15) Agreement that people with obesity are pleasant to look at	Strongly agree	41	9.9
	Moderately agree	22	5.3
	Slightly agree	26	6.3
	Neither agree nor disagree	194	46.9
	Slightly disagree	40	9.7
	Moderately disagree	28	6.8
	Strongly disagree	63	15.2
(UMB16) Agreement that special effort should ensure equal rights and privileges for people with obesity	Strongly agree	160	38.6
	Moderately agree	32	7.7
	Slightly agree	33	8
	Neither agree nor disagree	85	20.5
	Slightly disagree	19	4.6
	Moderately disagree	16	3.9
	Strongly disagree	69	16.7
(UMB17) Agreement that special effort should ensure equal salaries for people with obesity	Strongly agree	166	40.1
	Moderately agree	29	7
	Slightly agree	24	5.8
	Neither agree nor disagree	88	21.3
	Slightly disagree	22	5.3
	Moderately disagree	18	4.3
	Strongly disagree	67	16.2
(UMB18) Agreement that special effort should ensure equal educational opportunities for people with obesity	Strongly agree	176	42.5
	Moderately agree	30	7.2
	Slightly agree	19	4.6
	Neither agree nor disagree	81	19.6
	Slightly disagree	21	5.1
	Moderately disagree	19	4.6
	Strongly disagree	68	16.4
(UMB19) Agreement that special effort should ensure equal housing opportunities for people with obesity	Strongly agree	180	43.5
	Moderately agree	20	4.8
	Slightly agree	22	5.3
	Neither agree nor disagree	86	20.8
	Slightly disagree	18	4.3
	Moderately disagree	18	4.3
	Strongly disagree	70	16.9
(UMB20) Agreement that one tries to understand the perspective of people with obesity	Strongly agree	191	46.1
	Moderately agree	34	8.2
	Slightly agree	39	9.4
	Neither agree nor disagree	75	18.1
	Slightly disagree	23	5.6
	Moderately disagree	14	3.4
	Strongly disagree	38	9.2

Note. N=414. UMBFAT uses a 7-point scale (1=Strongly agree to 7=Strongly disagree). Items 1-6, 8, and 14 were reverse-coded for scoring, such that higher scores consistently indicate greater bias/fat phobia. The table shows the distribution of responses to the items *as stated* in the output (before reverse coding for indicated items).

Supplementary Table S6. Spearman Correlations Among Key Study Variables

Variable	1	2	3	4	5	6	7	8	9	10	11	12	13	14	15	16	17	18	19	20
1. Experienced Stigma	1																			
2. Internalized Weight Bias	.38***	1																		
3. Health Conversations	**-.01**	**-.08**	**1**																	
4. Weight Conversations	**.01**	**.24*****	**.25*****	**1**																
5. Comments: Own Weight	.06	.11*	.32***	.42***	1															
6. Comments: Others' Weight	.12*	.11*	.11*	.27***	.38***	1														
7. Comments: Diet/PA	.02	-.04	.28***	.21***	.55***	.34***	1													
8. AFA Total	.10*	.36***	.06	.04	.12*	.21***	.12*	1												
9. AFA: Dislike	.09	.30***	.02	.04	.11*	.25***	.07	.83***	1											
1. AFA: Fear of Fat	.18***	.48***	.07	.07	.11*	.15**	.09	.81***	.55***	1										
11. AFA: Willpower	.01	.10*	.11*	-.03	.07	.10*	.17**	.73***	.36***	.45***	1									
12. Total UMB Fat	-.13*	.04	**-.05**	.09	.03	.14**	-.01	.16**	.24***	.05	.06	1								
13. UMB: Neg. Judgment	-.03	.05	.02	.10*	0	.14**	-.01	.15**	.21***	.08	.04	.63***	1							
14. UMB: Distance	-.13**	.07	-.08	.14**	.06	.15**	0	.18***	.26***	.08	.06	.76***	.49***	1						
15. UMB: Attraction	-.08	.08	.07	-.01	.14**	.17***	.13**	.30***	.29***	.19***	.25***	.49***	.11*	.44***	1					
16. UMB: Equal Rights	-.05	.03	-.06	.03	-.04	.02	-.05	0	.05	-.05	-.03	.66***	.09	.26***	.12*	1				
17. Parental SRH	-.14**	-.36***	.11*	-.08	-.04	.01	.11*	-.14**	-.08	-.20***	-.08	-.03	-.09	-.05	-.07	.02	1			
18. Parental Chronic Morbidity	-.20***	-.17**	-.06	-.08	-.08	.03	-.06	-.07	-.02	-.17**	-.03	-.02	-.12*	-.03	-.05	.03	.42***	1		
19. Parental BMI	.23***	.42***	-.10*	.17***	.03	-.03	-.05	.05	-.04	.20***	-.01	.01	.03	-.03	-.07	.05	-.21***	-.17**	1	
20. Child's Age	-.09	-.08	.05	.17***	.16**	.05	0	-.12*	-.04	-.15**	-.12*	-.02	-.07	.02	.03	-.03	-.05	.05	.02	1
21. Child BMI Percentile	.12*	.21***	-.01	.50***	.13**	.05	-.06	-.02	-.02	.05	-.10*	.02	.09	.07	-.13**	0	-.14**	-.1	.26***	-.01

Note. PA = Physical Activity; AFA = Antifat Attitudes Questionnaire; BMI = Body Mass Index; UMBFAT = Universal Measure of Bias (Fat Phobia Scale); SRH = Self-Rated Health. N varies slightly for correlations involving SRH (n=402), Morbidity (n=364), and Child BMI Percentile (n=405) due to missing data. Coefficients are Spearman's rho (rs). * $p < .05$. ** $p < .01$. *** $p < .001$.

Supplementary Table S7. Predictors of Parental Weight and Health Communication

Predictor Variable	B	SE B	β	t	p
**Dependent Variable: Weight Conversations**					
Overall Model: $R2$=.331, Adj. $R2$=.306, $F(14, 369) = 13.04$, $p < .001$					
Block 1 ($\Delta R2$=.283, $p < .001$); Block 2 ($\Delta R2$=.048, $p < .001$)					
**Block 1: Covariates**					
Parent Age Group (ref. >40 yrs)	-0.045	0.088	-0.023	-0.514	0.608
Parent Gender (ref. Female)	-0.219	0.156	-0.062	-1.409	0.16
Parent Education (ref. Lower)	-0.019	0.113	-0.008	-0.165	0.869
Parent Marital Status (ref. Other)	-0.203	0.141	-0.064	-1.44	0.151
Multiple Children (ref. One Child)	0.088	0.088	0.044	1.002	0.317
Parent SRH (ref. Poor/Acceptable)	0.133	0.11	0.056	1.208	0.228
Parent BMI	0.003	0.009	0.018	0.369	0.713
Child Age (years)	0.1	0.025	0.179	3.929	<.001
Child Gender (ref. Female)	0.055	0.085	0.028	0.645	0.519
Child BMI Percentile	0.013	0.001	0.436	9.405	<.001
**Block 2: Main Predictors**					
Experienced Weight Stigma (ref. No)	-0.178	0.09	-0.092	-1.974	0.049
Weight bias internalization	0.197	0.044	0.251	4.501	<.001
Anti-Fat Attitudes Score	-0.018	0.032	-0.028	-0.576	0.565
UMBFAT Score	0.004	0.002	0.078	1.763	0.079
**Dependent Variable: Comments about Own Weight**					
Overall Model: $R2$=.125, Adj. $R2$=.092, $F(14, 369) = 3.77$, $p < .001$					
Block 1 ($\Delta R2$=.102, $p < .001$); Block 2 ($\Delta R2$=.023, $p = .045$)					
**Block 1: Covariates**					
Parent Age Group (ref. >40 yrs)	0.028	0.091	0.016	0.306	0.76
Parent Gender (ref. Female)	-0.243	0.162	-0.075	-1.495	0.136
Parent Education (ref. Lower)	0.449	0.118	0.198	3.801	<.001
Parent Marital Status (ref. Other)	-0.203	0.147	-0.071	-1.383	0.167
Multiple Children (ref. One Child)	0.077	0.092	0.042	0.841	0.401
Parent SRH (ref. Poor/Acceptable)	-0.088	0.115	-0.04	-0.761	0.447
Parent BMI	-0.006	0.009	-0.033	-0.584	0.56
Child Age (years)	0.107	0.027	0.21	4.030	<.001
Child Gender (ref. Female)	0.019	0.088	0.011	0.217	0.829
Child BMI Percentile	0.003	0.001	0.099	1.861	0.064
**Block 2: Main Predictors**					
Experienced Weight Stigma (ref. No)	-0.002	0.094	-0.001	-0.027	0.979
Weight bias internalization	0.096	0.046	0.134	2.108	0.036
Anti-Fat Attitudes Score	0.041	0.033	0.068	1.216	0.225
UMBFAT Score	0.001	0.002	0.012	0.235	0.814
**Dependent Variable: Comments about Others' Weight**					
Overall Model: $R2$=.117, Adj. $R2$=.084, $F(14, 369) = 3.50$, $p < .001$					
Block 1 ($\Delta R2$=.036, $p = .190$); Block 2 ($\Delta R2$=.082, $p < .001$)					
**Block 1: Covariates**					
Parent Age Group (ref. >40 yrs)	-0.051	0.092	-0.029	-0.553	0.581
Parent Gender (ref. Female)	0.082	0.164	0.025	0.501	0.617
Parent Education (ref. Lower)	0.194	0.119	0.086	1.631	0.104
Parent Marital Status (ref. Other)	-0.263	0.148	-0.091	-1.78	0.076
Multiple Children (ref. One Child)	0.166	0.093	0.09	1.788	0.075
Parent SRH (ref. Poor/Acceptable)	0.034	0.116	0.016	0.296	0.767
Parent BMI	-0.02	0.01	-0.117	-2.1	0.036
Child Age (years)	0.038	0.027	0.074	1.414	0.158
Child Gender (ref. Female)	0.054	0.089	0.03	0.606	0.545
Child BMI Percentile	0.002	0.001	0.067	1.266	0.206
**Block 2: Main Predictors**					

Experienced Weight Stigma (ref. No)	0.186	0.095	0.105	1.964	0.05
Weight bias internalization	0.08	0.046	0.112	1.751	0.081
Anti-Fat Attitudes Score	0.103	0.034	0.172	3.067	0.002
UMBFAT Score	0.005	0.002	0.113	2.207	0.028
**Dependent Variable: Comments about Diet/Exercise**					
Overall Model: R2=.074, Adj. R2=.039, F(14, 369) = 2.11, p = .011					
Block 1 (ΔR2=.060, p = .009); Block 2 (ΔR2=.014, p = .243)					
**Block 1: Covariates**					
Parent Age Group (ref. >40 yrs)	0.088	0.092	0.05	0.952	0.342
Parent Gender (ref. Female)	-0.084	0.163	-0.027	-0.514	0.607
Parent Education (ref. Lower)	0.436	0.119	0.197	3.674	<.001
Parent Marital Status (ref. Other)	0.022	0.148	0.008	0.148	0.883
Multiple Children (ref. One Child)	-0.056	0.092	-0.031	-0.607	0.544
Parent SRH (ref. Poor/Acceptable)	0.075	0.116	0.035	0.645	0.519
Parent BMI	-0.008	0.009	-0.051	-0.888	0.375
Child Age (years)	0.035	0.027	0.07	1.307	0.192
Child Gender (ref. Female)	0.068	0.089	0.039	0.766	0.444
Child BMI Percentile	-0.001	0.001	-0.045	-0.818	0.414
**Block 2: Main Predictors**					
Experienced Weight Stigma (ref. No)	0.016	0.094	0.009	0.167	0.867
Weight bias internalization	0.027	0.046	0.039	0.598	0.55
Anti-Fat Attitudes Score	0.061	0.034	0.104	1.819	0.07
UMBFAT Score	-0.001	0.002	-0.032	-0.615	0.539
**Dependent Variable: Health Conversations**					
Overall Model: R2=.080, Adj. R2=.045, F(14, 369) = 2.30, p = .005					
Block 1 (ΔR2=.074, p = .001); Block 2 (ΔR2=.006, p = .663)					
**Block 1: Covariates**					
Parent Age Group (ref. >40 yrs)	-0.04	0.075	-0.028	-0.528	0.598
Parent Gender (ref. Female)	-0.086	0.133	-0.033	-0.646	0.519
Parent Education (ref. Lower)	0.355	0.097	0.196	3.662	<.001
Parent Marital Status (ref. Other)	0.08	0.121	0.035	0.66	0.51
Multiple Children (ref. One Child)	0.001	0.076	0	0.007	0.994
Parent SRH (ref. Poor/Acceptable)	0.257	0.095	0.147	2.716	0.007
Parent BMI	-0.017	0.008	-0.122	-2.134	0.034
Child Age (years)	0.037	0.022	0.091	1.714	0.087
Child Gender (ref. Female)	-0.005	0.072	-0.004	-0.069	0.945
Child BMI Percentile	0.001	0.001	0.046	0.852	0.395
**Block 2: Main Predictors**					
Experienced Weight Stigma (ref. No)	-0.054	0.077	-0.038	-0.7	0.485
Weight bias internalization	0.047	0.037	0.082	1.252	0.212
Anti-Fat Attitudes Score	0.007	0.027	0.014	0.246	0.806
UMBFAT Score	-0.001	0.002	-0.027	-0.521	0.603

Note. B = unstandardized regression coefficient; SEB = Standard Error of B; β = standardized regression coefficient; BMI = Body Mass Index; UMBFAT = Universal Measure of Bias (Fat Phobia Scale); SRH = Self-Rated Health. ref. indicates the reference category for dummy coded variables. Significance level set at p<.05. All analyses based on N=384 due to listwise deletion of missing data. Final model statistics (R2, Adj. R2, F-test) are reported for the model including both blocks. Block statistics report the change in R2 (ΔR2) and the significance of that change when adding predictors in that block. Covariates were entered in Block 1, Main Predictors in Block 2.
